# *Lactobacillus acidophilus* alleviates slow transit constipation by modulating 5-HT pathway and gut microbial composition

**DOI:** 10.3389/fnut.2026.1775405

**Published:** 2026-03-16

**Authors:** Yunhe Fan, Hao Qin, Jiayao Liu, Mureed Abbas, Chuanli Yang, Haixia Cheng, Xiushan Dong

**Affiliations:** 1 Department of General Surgery, Shanxi Bethune Hospital, Shanxi Academy of Medical Sciences, Third Hospital of Shanxi Medical University, Tongji Shanxi Hospital, Taiyuan, China; 2 Shanxi Bethune Hospital, Shanxi Academy of Medical Sciences, Tongji Shanxi Hospital, Third Hospital of Shanxi Medical University, Taiyuan, China; 3 Modern Research Center for Traditional Chinese Medicine, The Key Laboratory of Chemical Biology and Molecular Engineering of Ministry of Education, Shanxi University, Taiyuan, China

**Keywords:** 5-hydroxytryptamine, fecal microbiota transplantation, gut microbiota, *Lactobacillus acidophilus*, slow transit constipation

## Abstract

**Introduction:**

Slow transit constipation (STC) is a chronic disease characterized by delayed intestinal transit and weakened spontaneous contractions of colonic smooth muscle. Current pharmacological treatments are often associated with adverse effects, highlighting the need for safe and more effective therapeutic strategies. This study investigated the potential role of *Lactobacillus acidophilus* (*L. acidophilus*) in regulating intestinal motility and alleviating STC, as well as the underlying mechanism.

**Methods:**

A humanized mouse model was established by intragastric administration of fecal bacterial suspension from STC patients on alternate days, in order to evaluate the effect of *L. acidophilus* on constipation. The regulatory effect of *L. acidophilus* on intestinal motility was evaluated using defecation parameters. Colon histopathology was assessed by hematoxylin-eosin (H&E) staining. Immunohistochemistry (IHC), RT-qPCR, ELISA, and *in vitro* cell experiments were performed to examine the inflammatory cytokine levels and changes in the 5-hydroxytryptamine (5-HT) signaling pathway. In addition, metagenomic sequencing was used to analyze changes in the intestinal microbial community.

**Results:**

The results showed *L. acidophilus* treatment significantly enhanced intestinal peristalsis and maintained the intestinal barrier by up-regulating *Occludin* expression and down-regulating inflammatory cytokines, including *TNF-α* and *IL-1β*, thereby suppressing inflammatory responses. Both *in vivo* and *in vitro* experiments showed that *L. acidophilus* affected the synthesis and release of 5-HT by regulating the expression of *TPH1* and the mechanosensitive ion channel *Piezo1*. Additionally, *L. acidophilus* reshaped the intestinal microbial community structure and altered the inter-bacterial interaction network, which was closely associated with improved intestinal motility.

**Conclusion:**

Our current research reveals that constipation symptoms by *L. acidophilus* through the gut microbiota composition, intestinal barrier, and the 5-HT signaling pathway. These findings provide a strong theoretical basis for the development of *L. acidophilus* as a potential therapeutic strategy for the treatment of STC.

## Introduction

Slow transit constipation (STC) is a subtype of functional constipation characterized by impaired colonic motility, resulting in delayed fecal transit and prolonged defecation time. Clinically, STC presents with infrequent bowel movements, difficulty in defecation, and a sense of incomplete evacuation (1, 2). Epidemiological studies indicate that approximately 37.9 to 55% of patients experience STC (3, 4). With the changes in dietary structure and lifestyle, the incidence of STC has shown a progressive annual increase with alarming trend towards younger age groups. The prevalence of constipation in the general population is estimated at around 20% (5) with STC incidence rising with age, and significantly higher among females (4). In the early stages, most patients do not pay sufficient attention and do not receive standardized diagnosis and treatment, leading to disease progression, chronicity and reduced treatment efficacy.

STC not only manifests as gastrointestinal discomforts such as abdominal distension and loss of appetite but also contributes to extragastrointestinal symptoms such as anxiety and depression, which seriously affect the quality of life. Although oral laxatives are used to treat constipation, prolonged use may disrupt intestinal microbiota balance and impair intestinal nerves and smooth muscle function, ultimately reducing intestinal sensitivity (6). Consequently, discontinuation of medication often results in symptom relapse or worsening constipation severity.

Accumulating evidence suggests that probiotics play a beneficial role in relieving constipation. Probiotics are active micro-organisms that colonize the host and alter the composition of a certain part of the host’s flora. Numerous studies have demonstrated that probiotics can alleviate constipation in various ways, including altering the composition of the intestinal flora, improving intestinal environment, and maintaining microbial balance (6, 7). Lactic acid bacteria (LAB) are one of the most common groups of probiotics naturally inhabiting the gastrointestinal tract and are also widely present in fermented foods (8). Various LAB strains possess proven probiotic properties and have been shown to provide diverse physiological benefits to the host (9, 10). Several studies have shown that certain strains of *lactobacillus* can improve intestinal function and relieve constipation (6, 11, 12). *Lactobacillus plantarum* PS128 enhances intestinal peristalsis, mucin production and serotonin signal transduction, thereby exerting a laxative effect in mice (13). Clinical trials have also shown that supplementation of *Bifidobacteria* spp. significantly increases weekly bowel movement frequency and improves stool consistency in patients with chronic constipation (14).

*Lactobacillus acidophilus*, which was first isolated from infant feces in 1900, is a non-spore-forming Gram-positive bacterium belonging to the genus *Lactobacillus* with the family *Lactobacillaceae*. It is recognized as an important intestinal probiotic closely associated with human health (15). *L. acidophilus* exhibits a broad range of biological functions, including enhancing immunomodulatory, nutritional, regulating the balance of intestinal flora, antioxidant, anti-carcinogenic, and cholesterol lowering effects (16–19). Previous studies have demonstrated that *L. acidophilus* can regulate the intestinal movement in zebrafish (2). Moreover, a probiotic mixture containing *L. acidophilus* have been shown to alleviate constipation symptoms in mice (20). Although these findings indicate the potential of *L. acidophilus* in regulating intestinal motility, its underlying molecular mechanism remains poorly understood. Therefore, the present study aimed to elucidate the regulatory effect of *L. acidophilus* on intestinal motility by evaluating fecal parameters and intestinal peristalsis in constipated mice, and to further investigate its involvement in the 5-HT signaling pathway.

## Materials and methods

### Preparation of *Lactobacillus acidophilus* B636 culture

The bacterial strain was purchased from BeNa Culture Collection (BNCC). After receiving the bacteria, the culture was inoculated into the MRS medium and cultured in an anaerobic incubator at 37 °C for 12 h. When the bacterial concentration reached approximately 10^10^ colony forming units (CFUs)/mL, the bacteria were stored at 4 °C for subsequent use. For cell-based assay the bacterial supernatant was obtained by centrifuging the refrigerated culture at 3000 rpm for 15 min, followed by filtration of supernatant through a 0.22 μm membrane filter.

### Animals and cells

Six-week-old adult female C57BL/6J mice were obtained from the Charles River (Beijing, China). Animals were placed in a temperature-controlled (22 ± 2 °C) and humidity-controlled (55–65%) facility at the Medical Experiment Center of Shanxi Bethune Hospital. Mice were maintained under a 12 h light/12 h dark cycle with free access to standard laboratory feed and tap water.

QGP-1 cell line was purchased from COBIOER (Nanjing, China). This cell line is a well-established and internationally recognized *in vitro* model for studying EC cell biology and the mechanisms of 5-HT synthesis and secretion. The cells were cultured in RPMI 1640 medium (Thermo Fisher Scientific, Shanghai, China), supplemented with 10% fetal bovine serum, and maintained in a humidified incubator at 37 °C with 5% CO_2._

### Experimental group design

**Figure 1:**
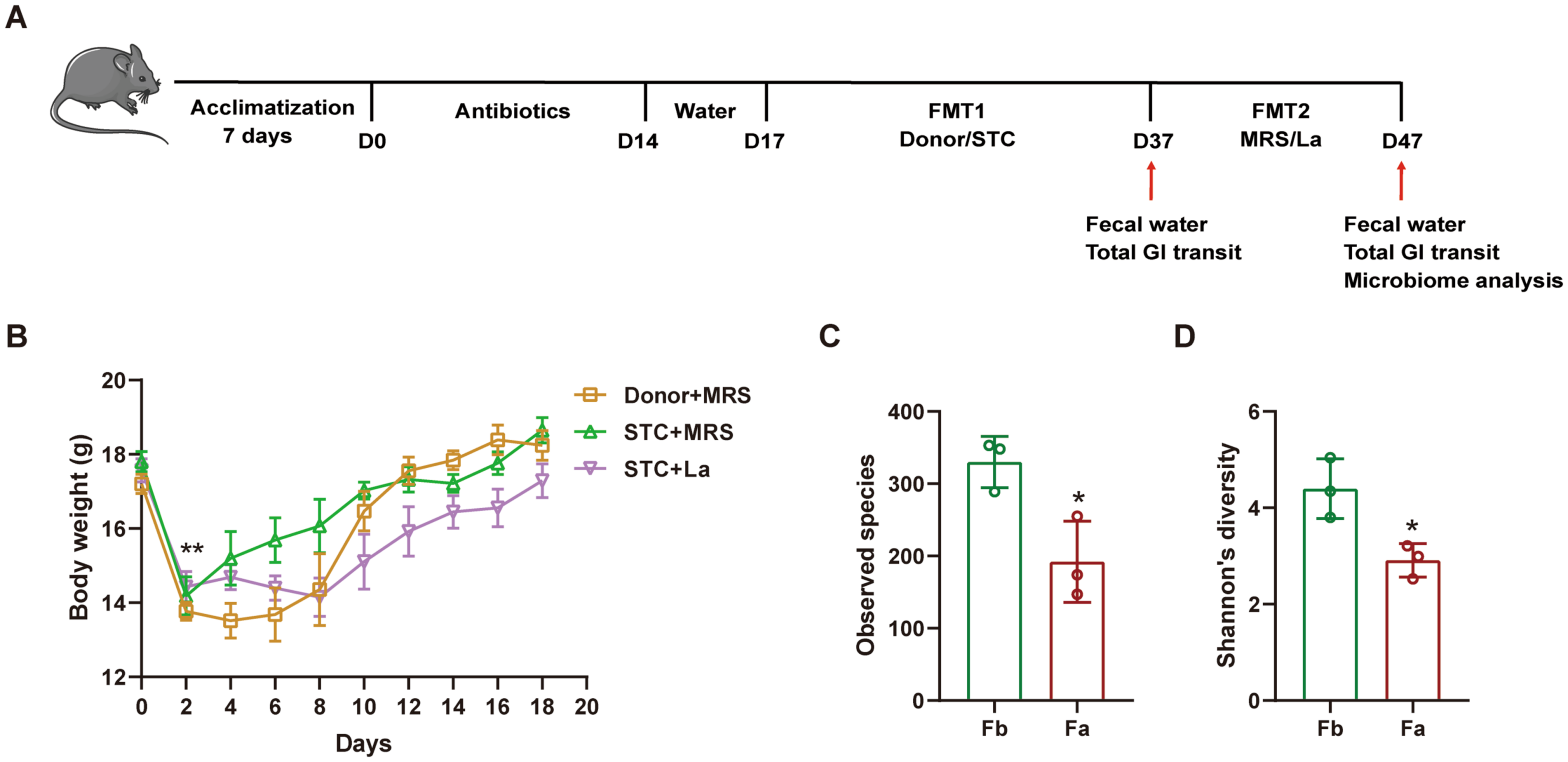
The experimental design **(A)** animal experimental flowchart. **(B)** Body weight. The alpha diversity of microbiomes identified in mice on **(C)** observed species and **(D)** Shannon’s diversity. Fb, before FMT; Fa, after FMT. *p < 0.05, *n* = 6.

The experimental design is illustrated in Figure 1A. Following a one-week acclimatization period, mice were randomly divided into three groups: Control group (Donor + MRS), Model group (STC + MRS) and *L. acidophilus* group (STC + La). All mice were received an antibiotic cocktail (consisting ampicillin [1 g/L], vancomycin [0.5 g/L], neomycin [1 g/L], and metronidazole [1 g/L]) in their drinking water supplemented with 10% sucrose for 2 weeks to deplete the gut microbiota. Afterward, mice had access to normal drinking water for 3 days. Subsequently, the first fecal microbiota transplantation (FMT) was performed. Mice in the Donor + MRS group received donors’ fecal bacterial suspension via oral gavage, whereas the STC + MRS and STC + La groups received fecal bacterial suspension derived from STC patients for 20 consecutive days. Afterwards, a second FMT was conducted: The Donor + MRS and STC + MRS groups received MRS medium by gavage, while the STC + La group received *L. acidophilus*. All animal procedures were approved by the Animal Care and Use Committee of Shanxi Bethune Hospital.

### Fecal moisture content test

After the first FMT (FMT1) and the second FMT (FMT2), each mouse was individually placed in a separate cage. Fresh fecal samples were collected from each mouse within 2 h. The wet weight of freshly collected fecal samples was recorded immediately. Samples were then dried in an oven at 60 °C to a constant weight. The fecal moisture content (%) was calculated as follows: (*wet weight − dry weight*)*/wet weight × 100* (21).

### Measurement of total gastrointestinal transit time (TGTT)

To assess TGTT, mice were fasted for 6 h and then gavaged with 0.3 mL of a non-absorbable dye solution (6% carmine red in 0.5% methylcellulose). The interval between gavage and the excretion of the first red-stained fecal pellet was documented as TGTT. During the measurement, mice were housed in separate cages lined with white filter paper to enhance visualization of excreted dye (22).

### Histopathology and immunohistochemistry

The colon tissues of each group of mice were dissected and placed separately into different 1.5 mL EP tubes. Samples were fixed in 4% paraformaldehyde, followed by standard dehydration, paraffin embedding and sectioning at 5 μm. The sections were stained with H&E, dehydrated and mounted with neutral mounting medium. Histopathological changes were examined using an OLYMPUS BX51 microscope.

Immunohistochemistry (IHC) was performed to assess the localization and relative expression level of TNF-α. Paraffin-embedded mouse colon sections underwent antigen retrieval by pressure heating in citrate buffer (pH 6.0). After which, endogenous peroxidase activity was blocked by 3% hydrogen peroxide for 10 min. Sections were then incubated with 5% (w/v) bovine serum albumin for 25 min at room temperature for protein blocking. Primary antibody incubation against TNF-α (Proteintech, Wuhan, China) (60291-1-IG) (dilution 1:1000) was performed overnight at 4 °C. After washing, sections were incubated with a horseradish peroxidase-conjugated secondary antibody for 1 h at room temperature. Hematoxylin counterstaining, dehydration, clearing, and mounting were subsequently performed. Stained slides were visualized by light microscopy, and the IHC quantification was performed using ImageJ software.

### Expression analysis of related genes

Transcript levels of target genes were measured by reverse transcription quantitative PCR (RT-qPCR). Total RNA was extracted from mouse colon tissues or QGP-1 cells using the RNAiso Plus kit (Takara, Dalian, China) according to the manufacturer’s instructions. 1 μg of total RNA was reverse-transcribed into first-strand cDNA using M-MLV reverse transcriptase (TaKaRa, Dalian, China). Primers were designed using Primer Premier 5.0 software (Table 1).

**Table 1:** Primers for RT-qPCR.

Gene name	Species	Sequence of primers (5–3)
GAPDH	Mouse	F-ATGGGAAGCTTGTCATCAACG
		R-AAGACACCAGTAGACTCCACG
TNF-α	Mouse	F-CGGGCAGGTCTACTTTGGAG
		R-CAGGTCACTGTCCCAGCATC
IL-1β	Mouse	F-TGGGAAACAACAGTGGTCAG
		R-CCATCAGAGGCAAGGAGGAA
Occludin	Mouse	F-GCGGAAGAGGTTGACAGTCC
		R-ACTCCCCACCTGTCGTGTAG
TPH1	Mouse	F-CTGCGACATCAGCCGAGAA
		R-TGGTCGGCGTCAAGTTCG
Piezo1	Mouse	F-GCTGTCCTCACCCGAATCCA
		R-GATAGGGCAAGGCGAAGG
RPL22	Human	F-TGATTGCACCCACCCTGTAG
		R-GGTTCCCAGCTTTTCCGTTC
TPH1	Human	F-TGCAAAGGAGAAGATGAGAGAATTTAC
		R-CTGGTTATGCTCTTGGTGTCTTTC
Piezo1	Human	F-ACCTGCGTCATCATCGTGTG
		R-AGTTGGTGCTGTTGGGGAAG

RT-qPCR was performed in a Light Cycler^®^ 480II (Roche, Basel, Switzerland) using SYBR Green Real-time PCR Master Mix (Promega, Madison, WI, USA). The 20 μL reaction mixture comprised: 10 μL 2× SYBR Green PCR Master Mix, 0.8 μL of each primer (0.4 μmol/L), 8 μL 10-fold diluted cDNA template, and nuclease-free water. Thermal cycling conditions included: 95 °C for 60 s (initial denaturation); 40 cycles of 95 °C for 5 s (denaturation) and 55 °C for 31 s (annealing/extension). Amplification specificity was confirmed by melt curve analysis. Relative mRNA expression was calculated using the $2^{-\Delta c_{T}}$ method and normalized to an internal reference gene.

### Enzyme-linked immunosorbent assay (ELISA)

After FMT2 treatment, blood samples were collected from the retro orbital sinus into 1.5 mL centrifuge tubes. Samples were centrifuged at 4000 rpm for 20 min, after which the supernatant was transferred to fresh 1.5 mL tubes and stored at −80 °C for later use. The collected mouse serum samples were two-fold dilution with 1× PBS. Then, the 5-HT concentration in the serum was detected using commercial mouse 5-HT enzyme-linked immunosorbent assay kit (Ruixin Biotech, Guangzhou, China) according to the manufacturer’s instructions.

After 24 h of adding varying concentrations of bacterial supernatant, the culture media from QGP-1 cells were collected and centrifuged it at 4000 rpm for 20 min to remove cell debris. The commercial human 5-HT enzyme-linked immunosorbent assay kit (Ruixin Biotech, Guangzhou, China) was used to detect 5-HT in the supernatant of QGP-1 cells according to the manufacturer’s protocol. The absorbance of the final product was measured on a microplate at a wavelength of 450 nm.

### 16S rRNA gene sequencing and analysis

16S rRNA sequencing was performed to analyze intestinal microbiota of mice before and after antibiotic treatment, as described previously (23). Total genomic DNA was extracted from mice fecal samples using the QuickGene DNA tissue kit (Kurabo, Neyagawa, Japan) following the manufacturer’s protocol. The V3-V4 hypervariable regions of the bacterial 16S rRNA gene were amplified using primers 338F (5′-ACTCCTACGGGAGGCAGCAG-3′) and 806R (5′-GGACTACHVGGGTWTCTAAT-3′). Purified PCR amplicons were paired-end sequenced on an Illumina MiSeq platform following standard protocols. Raw sequences were demultiplexed, quality-filtered, and analyzed using QIIME2. Denoising, chimera removal, and amplicon sequence variant (ASV) inference were performed with DADA2. Taxonomic assignment was conducted against the SILVA database with a 97% similarity threshold. Alpha diversity indices (Shannon, Chao1) were calculated from rarefied ASV tables.

### Metagenomic sequencing and analysis

The gut microbiome of mice was investigated using the metagenomic shotgun sequencing method as described previously (24). Genomic DNA was extracted from fecal samples using the Magnetic Bead-Based Stool DNA Extraction Kit (BioTeKe, Beijing, chain). DNA libraries were constructed using the TruSeq Nano DNA Library Preparation Kit-Set (Illumina, USA) following the manufacturer’s instructions. Metagenome libraries were then sequenced on an Illumina NovaSeq 6000 platform with PE150 at Shanghai Biotree Biotech Co., Ltd. (Shanghai, China). Fastp software (v0.23.4) was used to remove the reads that contained adaptor contamination, low quality bases and undetermined bases. Then sequence quality was also verified using fastp. Quality filtered reads were first aligned to mice genome^1^ by using bowtie (v2.2) to filter out host contaminations. Then, the remaining reads were subjected to *de novo* assembly for each sample using MEGAHIT (v1.2.9) and used to assign microbial functions and taxonomy. Metaprodiga (v2.6.3) was used to predict the coding regions (CDS) of the assembled contigs, and CDS sequences of all samples were clustered using MMseq2 (v15-6f452) to obtain unigenes. DIAMOND (v0.9.14) was used to perform a taxonomic assessment of the microbiota based on the NR database. The wilcoxon rank-sum test was used to identify the differentially abundant species, and significances were declared at *p* < 0.05 and |log2-fold change| > 1. An assignment of microbial functions was done using the Kyoto Encyclopedia of Genes and Genomes (KEGG). Network analysis was performed using R 3.2.6 and Gephi v.0.9.2 software; *p* < 0.05, |*R*| > 0.6 were considered statistically significant.

### Statistical analysis

Statistical analyses were performed using Student’s *t*-test for comparisons between two groups. Data are presented as mean ± standard deviation (SD). Statistical significance was defined as *p < 0.05, **p < 0.01 and ***p < 0.001.

## Results

### Effect of *L. acidophilus* on fecal excretion parameters in STC mice

Following a 14-day antibiotic pretreatment regimen, constipation was induced in mice through FMT using samples obtained from STC patients. *L. acidophilus* was subsequently administered, and defecation related parameters were evaluated on days 37 and 47 to assess its therapeutic effects on STC-associated symptoms (Figure 1A). A significant reduction in body weight was observed in all mice on the second day after antibiotic administration (*p* < 0.01). Subsequently, the body weight of the mice gradually increased, indicating that the impact of antibiotics on body weight was transient and diminished over time (Figure 1B). Alpha diversity analysis demonstrated a significant reduction in gut microbial richness in antibiotic-treated mice compared to pre-treatment baselines (*p* < 0.05), suggesting that antibiotic intervention depleted a portion of the intestinal microbiota (Figures 1C,D).

**Figure 2:**
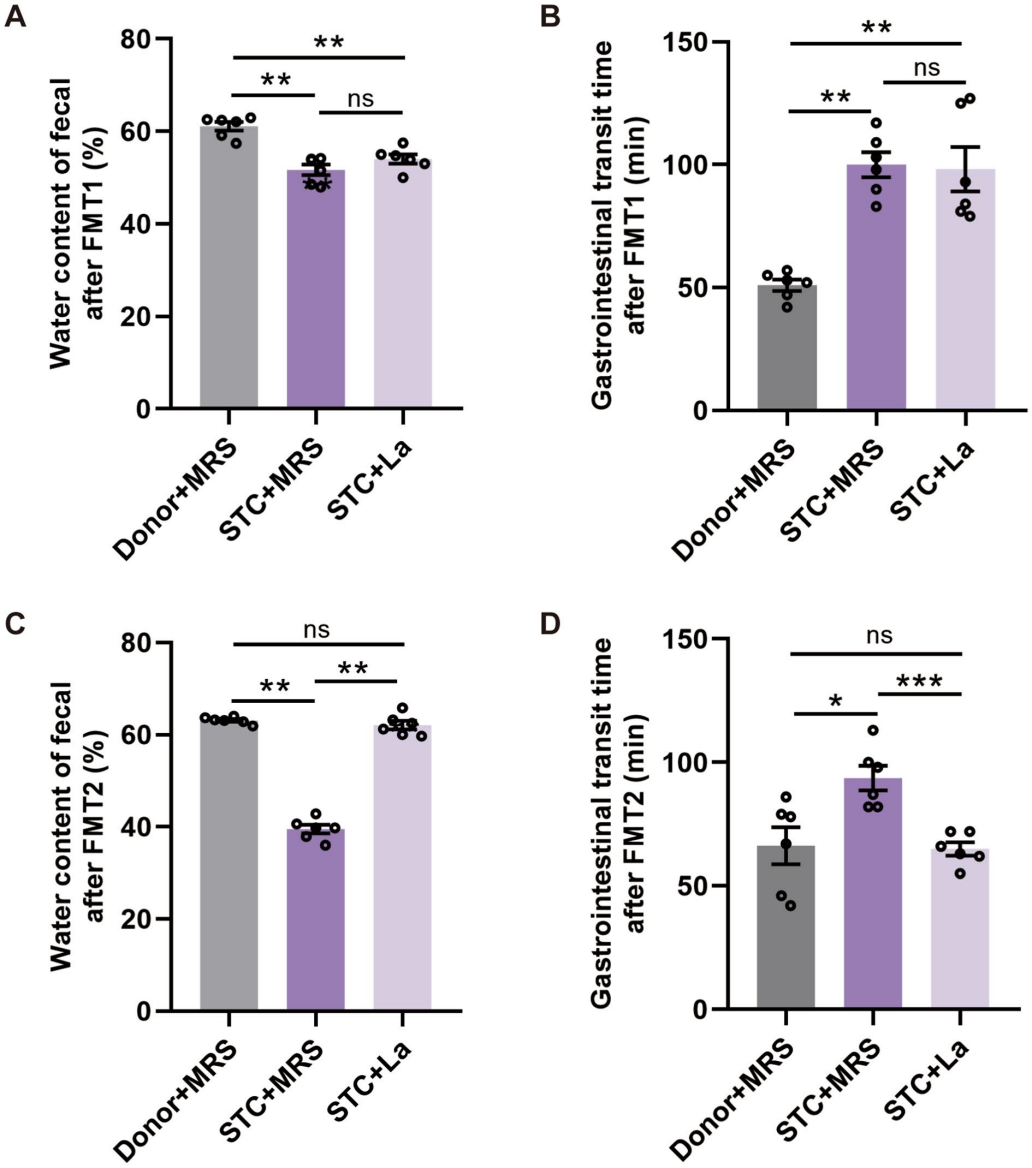
Effect of *L. acidophilus* on excretion parameters in mice. **(A)** Water content of fecal and **(B)** gastrointestinal transit time after FMT1. **(C)** Water content of fecal and **(D)** gastrointestinal transit time after FMT2. ^*^p < 0.05, **p < 0.01, ***p < 0.001, *n* = 6.

After FMT1, mice receiving STC patient-derived microbiota exhibited markedly altered defecation parameters compared to control group (Donor + MRS). These mice exhibited a significant reduction in fecal water content (*p* < 0.01) and a significant increase in TGTT (*p* < 0.01), indicating successful induction of constipation phenotypes by STC patients’ fecal microbiota (Figures 2A,B). Subsequently, the FMT2 was performed. The results showed that the mice treated with *L. acidophilus* had significantly different fecal parameters compared to the STC + MRS group. Specifically, the water content of the fecal was significantly increased (*p* < 0.01), while their TGTT was significantly decreased (*p* < 0.001) (Figures 2C,D).

### Effect of *L. acidophilus* on colonic histopathology in mice

**Figure 3:**
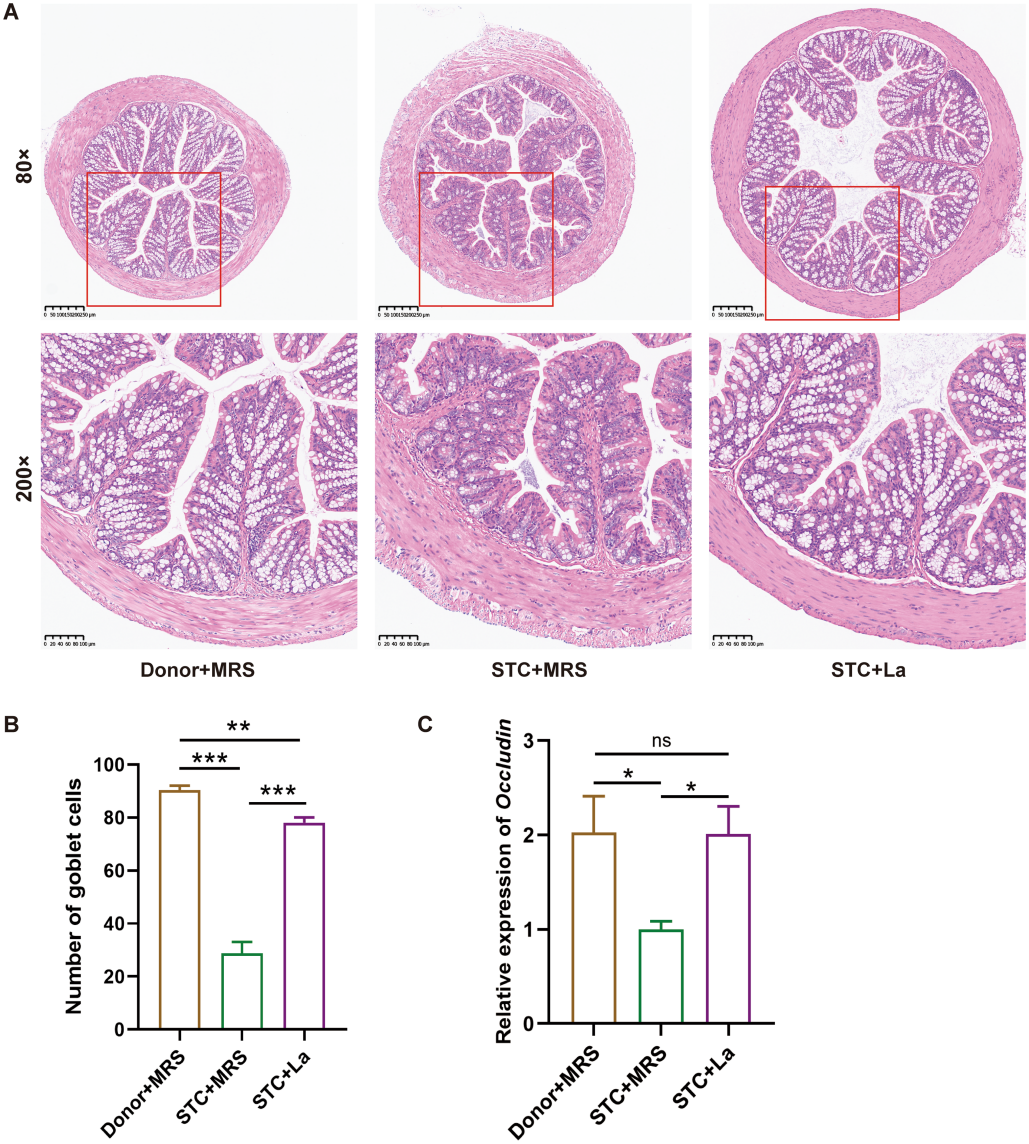
*Lactobacillus acidophilus* improves intestinal barrier damage. **(A)** Representative images of H&E staining of the colon tissue in each group. **(B)** The number of goblet cells in colon tissue from each group. **(C)** The relative expression of *Occludin* in colon sections. *p < 0.05, **p < 0.01, ***p < 0.001.

To evaluate the impact of *L. acidophilus* on colonic pathology, H&E staining was performed to examine the histological architecture of mouse colon. The results showed that in the control group, the structure of the colon tissue in mice was intact and clear, with orderly arranged goblet cells, and no observable pathological lesions. However, the STC + MRS group exhibited extensive intestinal mucosal layer necrosis, in which the necrotic intestinal glands were replaced by proliferating fibrous tissue, the structure of the mucosal layer disappeared, accompanied by diffuse inflammatory cell infiltration. Additionally localized enlargement of the muscle fiber gaps and loosened fiber arrangement were observed in the muscular layer. Following *L. acidophilus* treatment, the inflammatory infiltration was markedly reduced and the goblet cells appeared more regularly arranged (Figure 3A). The number of goblet cells in the colon tissue was calculated using the ImageJ software. The results showed that compared with the control group, the number of goblet cells in the STC + MRS group was significantly reduced (*p* < 0.001). Conversely, the STC + La group showed a significant increase in goblet cell numbers relative to STC + MRS group (*p* < 0.001) (Figure 3B). Further, expression of Occludin gene were examined using RT-qPCR as shown in Figure 3C. The results showed that compared with the Donor + MRS group, the mRNA level of *Occludin* in the STC + MRS group was significantly decreased (*p* < 0.05), and upon the addition of *L. acidophilus*, the expression of *Occludin* was significantly increased compared with the STC + MRS group (*p* < 0.05).

### Effect of *L. acidophilus* on inflammatory cytokine levels in mice

**Figure 4:**
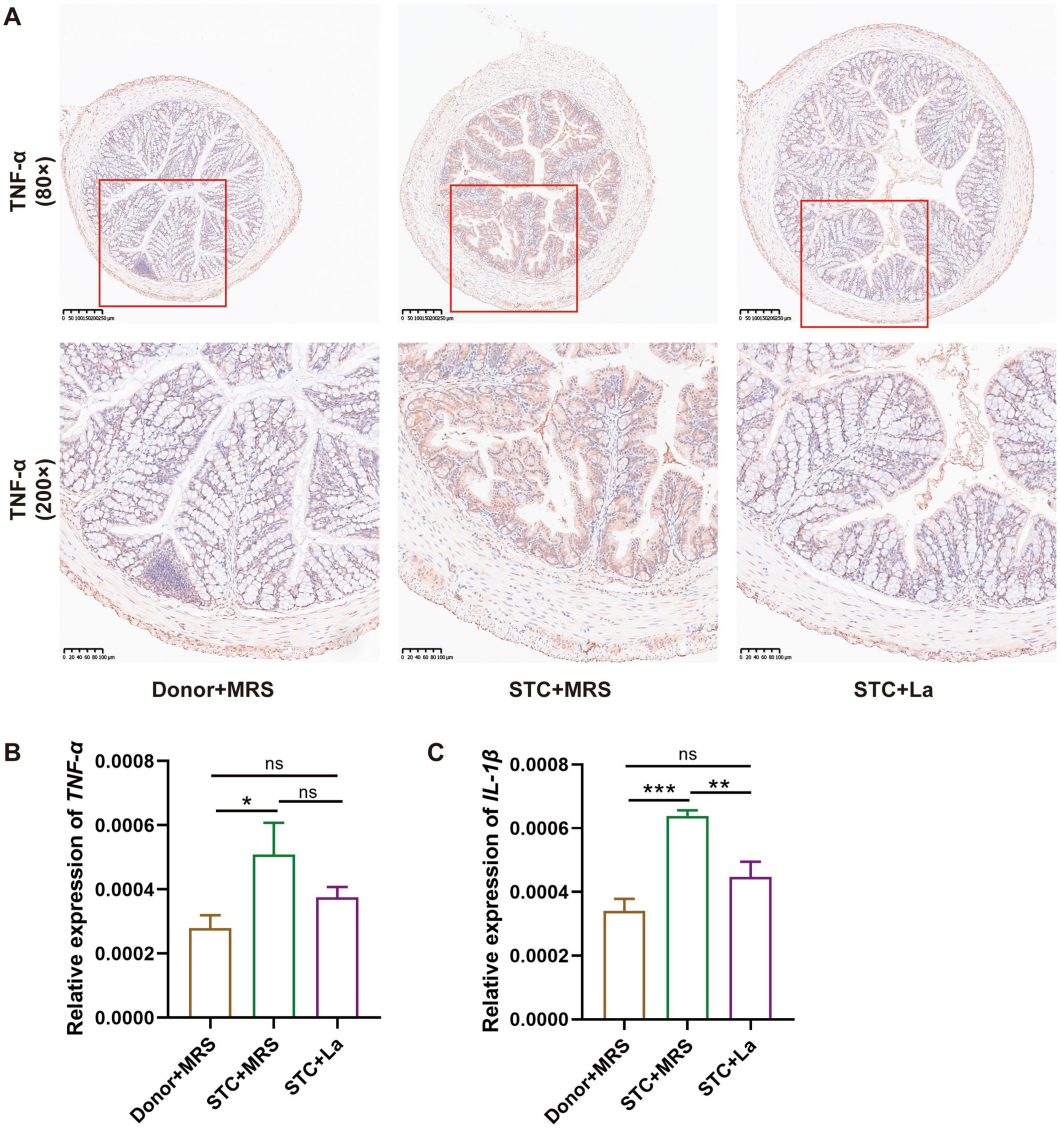
*Lactobacillus acidophilus* modulates the expression of inflammatory cytokines. **(A)** Immunohistochemical analysis of TNF-α protein expression in mouse colon tissue. **(B)** RT-qPCR analysis of *TNF-α* mRNA expression in colonic tissues. **(C)** RT-qPCR analysis of *IL-1β* mRNA expression in colonic tissues. *p < 0.05, **p < 0.01, ***p < 0.001.

IHC analysis demonstrated significantly elevated expression of inflammatory cytokine TNF-α in the colonic mucosa of the STC + MRS group compared with the control group, whereas no significant difference in TNF-α expression was observed between the STC + La and control group (Figure 4A). These observations were further validated by RT-qPCR analysis, which revealed significantly increased mRNA expression level of *TNF-α* and *IL-1β* in the STC + MRS group relative to the control group (*p* < 0.05; *p* < 0.01). In contrast, the expression levels of *TNF-α* and *IL-1β* in the STC + La group did not differ significantly from those in the control group (Figures 4B,C).

### Effect of *L. acidophilus* on 5-HT signal transduction pathway

Previous studies have shown that STC can affect the 5-HT signaling pathway (25, 26). Therefore, both *in vivo* and *in vitro* experiments were performed to investigate the regulatory effects of *L. acidophilus* on 5-HT signal transduction.

**Figure 5:**
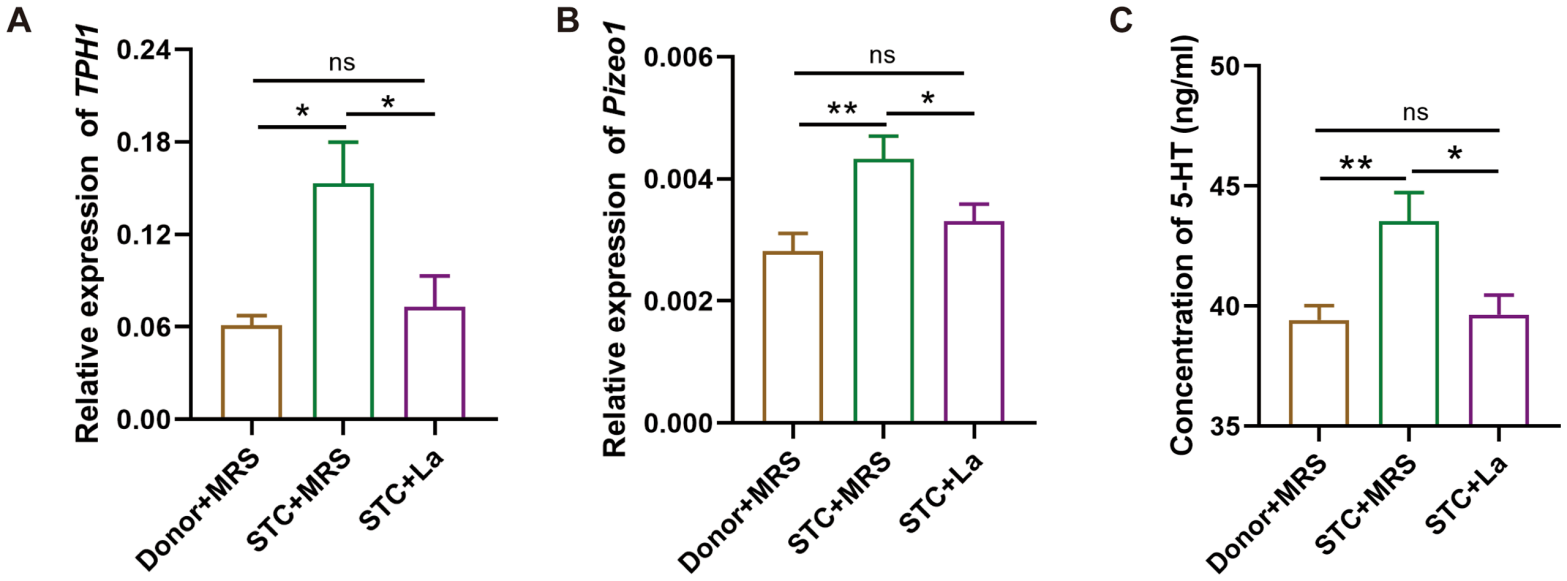
*Lactobacillus acidophilus* reduces 5-HT production by targeting *TPH1* and *Piezo1* in mice. **(A)** RT-qPCR analysis of *TPH1* mRNA expression in colonic tissues. **(B)** RT-qPCR analysis of *Piezo1* mRNA expression in colonic tissues. **(C)** Measurement of 5-HT levels in serum. *p < 0.05, **p < 0.01.

To assess the effect of *L. acidophilus* on the 5-HT signaling pathway *in vivo*, the expression of *tryptophan hydroxylase 1* (*TPH1*) and *Piezo1* in colonic tissues was quantified by RT-qPCR and the concentration of 5-HT in the serum was detected using ELISA. Compared with the control group, mice in the STC + MRS group exhibited significantly elevated expression of *TPH1* and *Piezo1* (*p* < 0.05; *p* < 0.01); in contrast, administration of *L. acidophilus* significantly reduced the expression of *TPH1* and *Piezo1* compared with the STC + MRS group (*p* < 0.05) (Figures 5A,B). Consistently, the concentration of 5-HT in the serum of mice in the STC + MRS group was significantly higher than in the control group (*p* < 0.05), whereas after adding *L. acidophilus*, 5-HT concentration was significantly decreased relative to STC + MRS group (Figure 5C).

**Figure 6:**
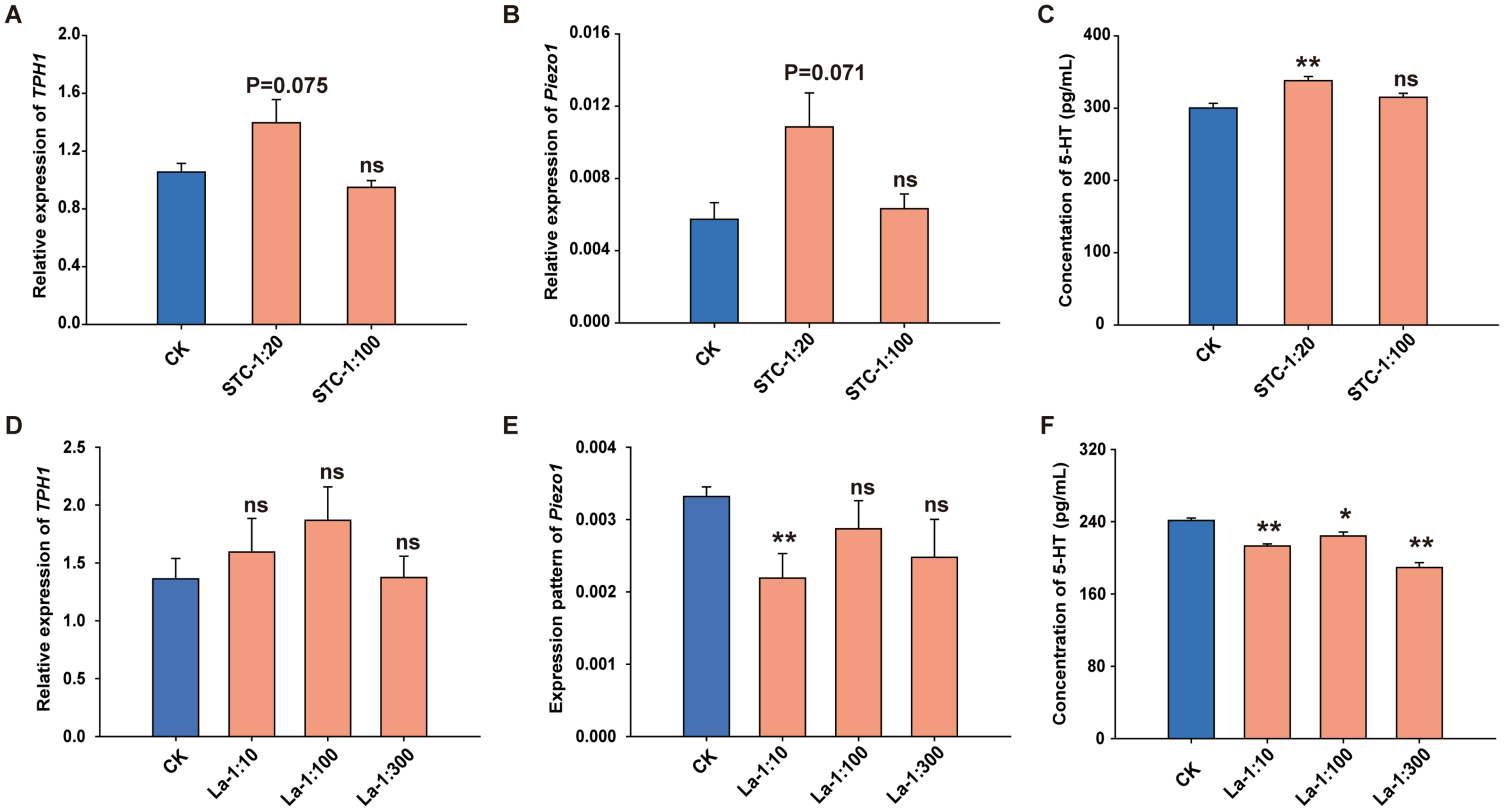
*Lactobacillus acidophilus* reduces 5-HT production by targeting *TPH1* and *Piezo1* in QGP-1 cells. Relative expression of **(A)** *TPH1* and **(B)** *Piezo1* after treated with STC patients’ fecal supernatant. **(C)** 5-HT content in the cell supernatant after treated with STC patients’ fecal supernatant. Relative expression of **(D)** *TPH1* and **(E)** *Piezo1* after treated with *L. acidophilus* supernatant. **(F)** The content of 5-HT in the cell supernatant after treated with *L. acidophilus* supernatant. *p < 0.05, **p < 0.01.

To further assess the regulatory effects of *L. acidophilus* on 5-HT signaling *in vitro*, QGP-1 cells were treated with supernatants of STC patients’ bacteria and *L. acidophilus, respectively,* at different concentrations. After 24 h of incubation, the transcript levels of *TPH1* and *Piezo1* were quantified by RT-qPCR, and secreted 5-HT levels in cell supernatants were measured by ELISA. Compared with the control group, after QGP-1 cells were incubated with the supernatant of STC patient cultures at a concentration of 1: 20, the expression levels of *TPH1* and *Piezo1* showed an upward trend (*p* = 0.075; *p* = 0.071) respectively (Figures 6A,B). The ELISA results also indicated that after the cells were treated with the supernatant of STC patient cultures at a concentration of 1: 20, the content of 5-HT in the cell culture medium significantly increased (*p* < 0.01) (Figure 6C). Conversely, after incubation of *L. acidophilus* solution with QGP-1, although the expression of *TPH1* showed no significant alteration compared to the control (Figure 6D), the expression of *Piezo1* was significantly down-regulated at 1: 10 (*p* < 0.01) (Figure 6E), and the concentration of 5-HT in the culture supernatants was significantly reduced across all tested *L. acidophilus* concentrations (*p* < 0.05, *p* < 0.01) (Figure 6F).

### *Lactobacillus acidophilus* improves gut microbiota dysbiosis in STC model mice

**Figure 7:**
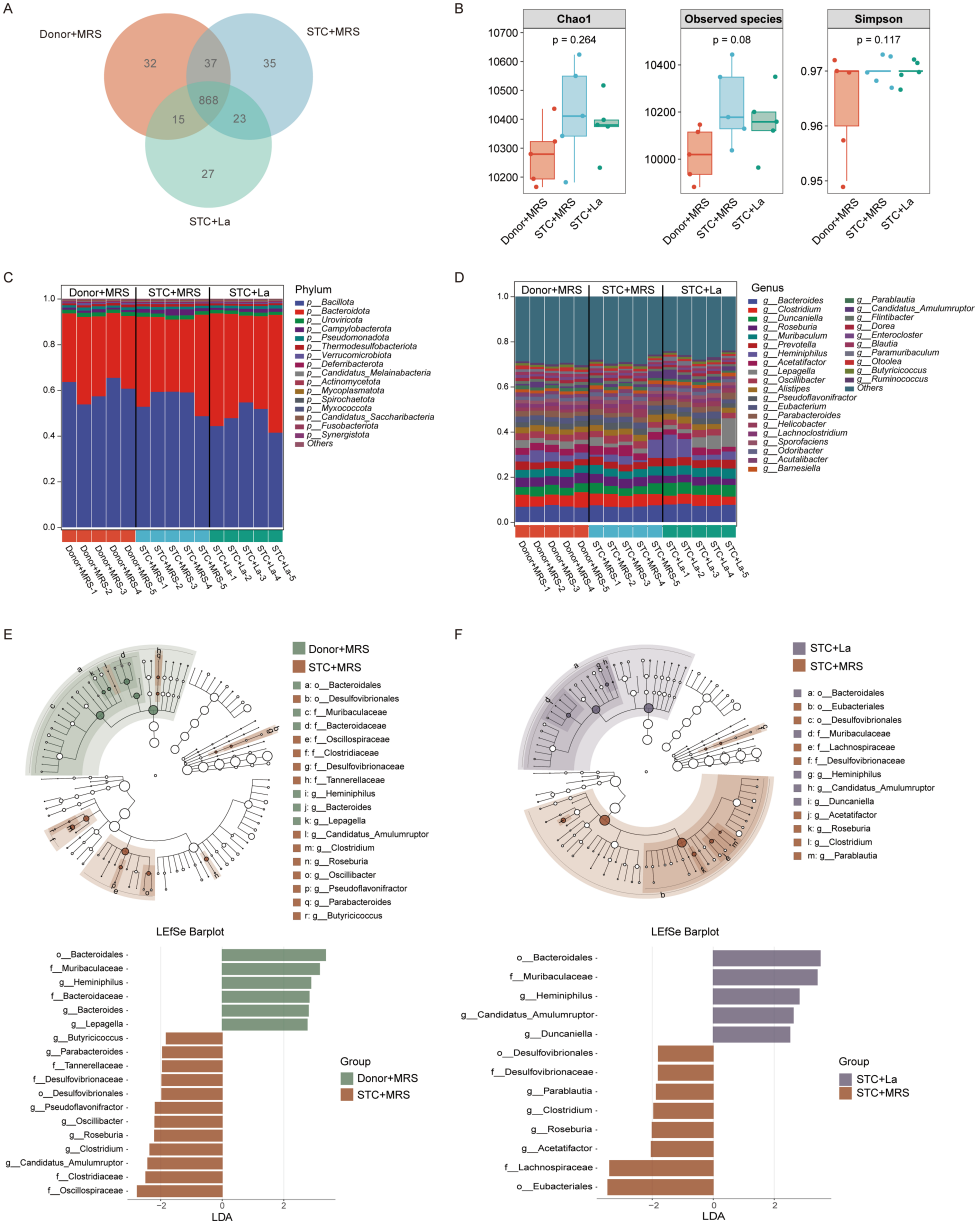
Effects of *L. acidophilus* on the composition of gut microbiota in constipation mice. **(A)** Venn diagram. **(B)** Comparison of α-diversity indices (Chao, observed species, and Simpson) among groups; **(C)** taxonomic distribution of bacterial communities at the phylum level; **(D)** taxonomic distribution of bacterial communities at the genus level; LEfSe analysis identifies the most differentially abundant bacterial taxa from phylum to species level between donor + MRS and STC + MRS groups **(E)**; and between STC + *La* and STC + MRS groups **(F)**.

Growing evidence indicates that the gut microbiota plays a critical role in maintaining intestinal health, and dysbiosis of the gut microbial community is closely associated with a range of gastrointestinal dysfunctions. To further investigate the effects of *L. acidophilus* on gastrointestinal motility, DNA was extracted from mouse fecal samples and subjected to metagenomic sequencing analysis. Venn diagram analysis at species level showed that the numbers of intestinal bacteria in the Donor + MRS, STC + MRS, and STC + La groups were 952, 963, and 923, respectively (Figure 7A). To evaluate disparities in gut microbiota diversity, alpha diversity analyses were conducted. The analysis revealed no statistically significant differences in the Chao1, Observed species, and Simpson indices (Figure 7B), demonstrating that overall microbial richness and diversity did not differ significantly among the groups.

To further characterize microbial community composition, taxonomic differences were analyzed at multiple levels. At the phylum level, *Bacillota* and *Bacteroidota* were the dominant phyla in all groups, collectively accounting for more than 90% of the total microbial abundance (Figure 7C). Compared with the STC + MRS group, the STC + La group exhibited a significant decrease in the relative abundance of *Bacillota* (STC + MRS group: 0.558 vs. STC + La group: 0.480, *p* = 0.043) and a significant increase in the abundance of *Bacteroidota* (STC + MRS group: 0.360 vs. STC + La group: 0.450, *p* = 0.038). At the genus level (Figure 7D), *L. acidophilus* intervention significantly reduced the relative abundances of *Clostridium* (STC + MRS group: 0.053 vs. STC + La group: 0.044, *p* = 0.016) and *Roseburia* (STC + MRS group: 0.040 vs. STC + La group: 0.033, *p* = 0.026), both belonging to *Bacillota* phylum, while increasing the relative abundance of *Muribaculum* (STC + MRS group: 0.037 vs. STC + La group: 0.042, *p* = 0.028), which is part of *Bacteroidota* phylum. These findings suggest that *L. acidophilus* alleviates constipation in mice, primarily through its association with *Bacillota* and *Bacteroidota* in the gut. Furthermore, Linear Discriminant Analysis (LDA) combined with Effect Size (LEfSe) analysis was used to identify differences in gut microbiota composition among groups. The results showed that the dominant microbiota in the Donor + MRS and STC + La groups included *o_Bacteroidales*, *f_Muribaculaceae*, and *g_Heminiphilus*, whereas the STC + MRS group was primarily characterized by *g_Roseburia*, *g_Clostridium*, and *f_Desulfovibrionaceae* (Figures 7E,F).

**Figure 8:**
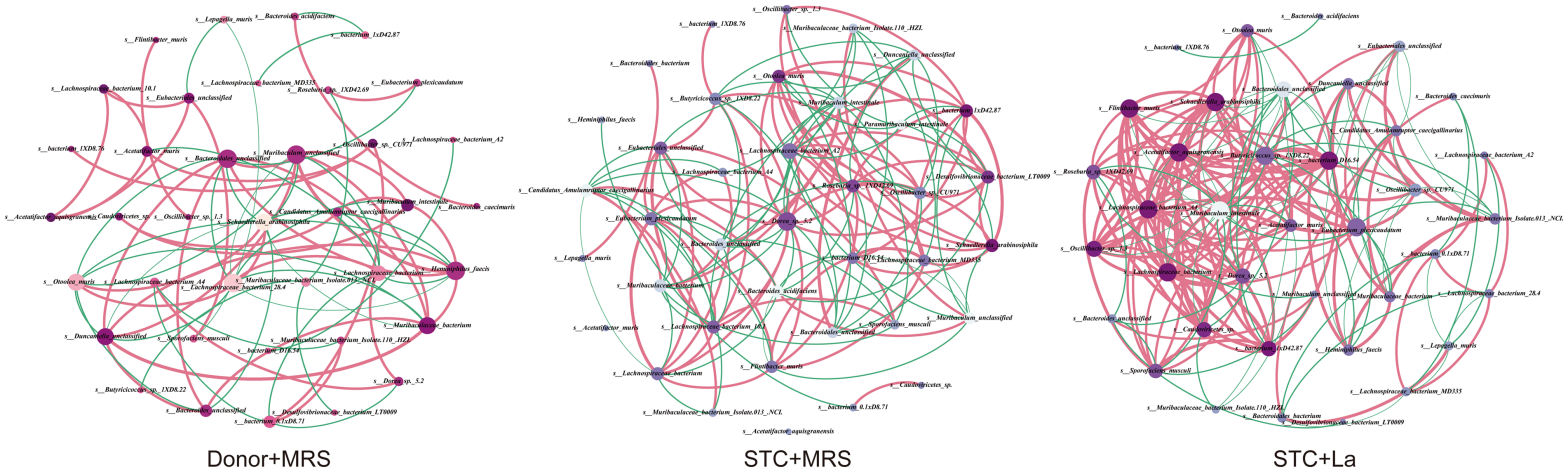
Co-occurrence networks of the top 38 species among different groups. Nodes represent species and their sizes indicate different relative abundance. The lines between nodes indicated the Spearman correlation (Spearman’s correlation greater than 0.6 or lower than −0.6), and the color of lines indicated the correlation coefficient (pink, positive; blue, negative).

Network correlation analyses were conducted in three experimental groups to evaluate the competitive associations within the intestinal microbial communities of the mice at the species level. The top 38 bacterial species with the highest relative abundances were selected for analysis, with each node representing a bacterium and node size corresponding to its relative abundance. Each line represents the interaction between bacteria, with pink lines indicating positive correlations and green lines indicating negative correlations. In the Donor + MRS group, a total of 110 connections were identified among the 38 nodes, of which 69.44% represented positive correlations, The STC + MRS group exhibited 172 connections, with 61.62% being positive correlations. Notably, the STC + La group displayed the highest number of interactions with 222 connections, 69.37% of which were positive correlations (Figure 8). Overall, the microbial co-occurrence network in the STC + La group was the most complex. Furthermore, comparative analysis of network topology revealed marked differences among the three groups in key parameters, including average degree, average path length and network density (Table 2).

**Table 2:** Network topology parameters for the 3 groups.

Network indices	Donor + MRS	STC + MRS	STC + La
Total nodes	38	38	38
Total edges	110	172	222
Positive edges (%)	69.44%	61.62%	69.37%
Negative edges (%)	30.56%	38.38%	30.63%
Average degree	5.789	9.053	11.684
Network diameter	10	4	5
Average path length	3.713	2.006	1.921
Density	0.156	0.245	0.316
Average clustering coefficient	0.752	0.673	0.828

## Discussion

STC is a common gastrointestinal disorder characterized by delayed colonic peristalsis and obstruction of intestinal contents excretion. This condition substantially affects patients’ physical and psychological well-being, as well as their overall quality of life, and may potentially lead to secondary gastrointestinal complications. Laxatives are commonly used for symptomatic relief; however, they may provide rapid improvement, long-term use can easily lead to an imbalance in the intestinal homeostasis, and can impair intestinal nerves and smooth muscles function, ultimately reducing the sensitivity of the intestine (6). Notably, discontinuation of laxative therapy often results in severe constipation with exacerbated symptoms. A large number of studies have shown that the gut microbiota plays a crucial role in maintaining intestinal homeostasis and peristalsis. Patients with STC generally have an imbalance in the intestinal microbiota, with a significant reduction in beneficial bacteria (such as *Bifidobacteria* and *Lactobacilli*), and an increase in the proportion of potentially pathogenic bacteria such as *Clostridium* and *Clostridium perfringens* (27). Numerous studies have demonstrated that probiotic supplementation can alleviate constipation by modulating gut microbiota composition. However, research has predominantly focused on multi-strain probiotic formulations, such as Leven PRO-CR and SynBalance SmilinGut, rather than on the effects of individual probiotic strains (20, 26). Recent clinical studies have reported significant improvement in constipation symptoms among patients following FMT, accompanied by a marked increase in *L. acidophilus* abundance as revealed by metagenomic analysis. These findings suggest a potential role for *L. acidophilus* in the regulation of intestinal motility (24). Although *L. acidophilus* is a well-established probiotic, and formulations containing *L. acidophilus* have been shown to alleviate constipation (28), it remains unclear whether *L. acidophilus* alone is sufficient to exert these effects. Therefore, in the present study, we systematically investigated the impact of single-strain *L. acidophilus* administration on intestinal motility using a mouse model of constipation and further explored the underlying molecular mechanisms involved.

Given the crucial role of gut microbiota in regulating intestinal motility, an antibiotic cocktail was administered in this study to deplete the indigenous intestinal microbes in mice. Although transient reduction in body weight was observed during antibiotic treatment, likely due to profound diarrhea induced by the treatment, 16S rRNA gene sequencing confirmed a significant reduction in gut microbial diversity following antibiotic administration. This approach is consistent with clinical practice where bowel cleansing using antibiotics is performed prior to FMT. Following antibiotic-mediated depletion of gut microbiota, a STC mouse model was established via FMT using fecal samples from patients with STC. This approach fundamentally differs from conventional constipation model induced by loperamide hydrochloride. Although the loperamide-induced model is widely adopted due to its operational simplicity and consistent induction of stable constipated phenotypes (29–31), it fails to adequately recapitulate female-specific constipation phenotypes and exhibits poor clinical relevance to the complex etiology of human constipation. Therefore, this study established a constipation mouse model using fecal microbiota from STC patients. This approach directly recapitulates the characteristic gut dysbiosis observed in constipated patients. Model mice successfully reproduced the microbial community structure of STC patients. Consequently, it provides a more physiologically relevant representation of the complete pathogenic cascade from microbial disruption to functional impairment. Furthermore, as this model is derived directly from human clinical samples, it exhibits superior predictive validity and enhanced translational potential for evaluating the efficacy of probiotic interventions.

Previous studies have reported that loperamide-induced constipation is associated with a significant reduction in gut microbial diversity (32, 33). However, our results showed that, compared with the Donor treatment group, the intestinal microbiota diversity of mice fed with fecal suspension from STC patients did not show significant change. We propose that the intestinal flora disorder in STC patients is not simply a decrease in microbial diversity, but rather a disruption in the balance between beneficial and pathogenic bacteria. Furthermore, network correlation analysis revealed that, compared to the Donor + MRS group, the bacterial interactions in the STC + MRS group were notably more complex, with a significant increase in negative correlations among bacterial species. Supplementation with *L. acidophilus* modified these inter-bacterial interactions, markedly enhanced positive correlations among bacterial taxa. These findings suggest that *L. acidophilus* may improve intestinal motility by remodeling gut microbial interaction networks rather than by simply increasing microbial diversity.

Among probiotics, *Lactobacillus* and *Bifidobacterium* have been extensively investigated for their ability to modulate the gut microbiota composition and intestinal function. Previous studies have shown that *Lactobacillus plantarum* promotes intestinal motility by regulating the 5-HT signaling pathway and enhancing mucus secretion (13), whereas *Bifidobacterium longum* alleviates constipation by improving intestinal barrier integrity (34). In this study, we demonstrated that administration of *L. acidophilus* significantly ameliorated constipation symptoms in mice, as evidenced by improved excretion parameters. Furthermore, HE staining revealed substantial structural damage to the intestinal tissue in STC mice, accompanied by significantly reduced expression of *Occludin* and a substantial upregulation of inflammatory factors (*TNF-α* and *IL-1β*). These findings are consistent with previous reports (32, 35). Growing evidence suggests that constipation is closely associated with dysfunction of the intestinal mucosal immune system (36). Constipated patients exhibit immune activation and a certain degree of mucosal inflammation (37). Activated immune cells release a range of inflammatory cytokines and neurotransmitters, which can disrupt the intestinal mucosal barrier, ultimately impairing gut sensation and motility. For example, interferon-γ (IFN-γ) has been shown to increase intestinal permeability by downregulating *Zonula Occludens-1* (*ZO-1*) expression and altering the distribution of tight junction proteins, such as *Occludin* (38). These mechanism provide a plausible explanation for our observations that STC mice showed significantly decreased *Occludin* expression and elevated levels of inflammatory factors in the colon. Importantly, *L. acidophilus* treatment increased *Occludin* expression and decreased expression of *TNF-α* and *IL-1β*, thereby improving intestinal barrier structure and ultimately alleviating constipation symptoms.

Furthermore, the 5-HT signal transduction pathway is also closely associated with the pathophysiology of constipation. As a key neurotransmitter and paracrine signaling molecule, 5-HT plays a crucial role in the gastrointestinal tract. Approximately 90% of 5-HT in the human body is synthesized by intestinal enterochromaffin cells (ECs) through the catalytic activity of TPH1 (39). Newly synthesized 5-HT is released into the intestinal lumen via ion channels. Studies have demonstrated that Piezo1 is a mechanosensitive ion channel expressed on ECs, capable of sensing mechanical stimuli (40, 41). Following 5-HT release, a portion of 5-HT is taken up into cells via Serotonin-selective reuptake transporter (SERT) and subsequently degraded, while the remaining fraction exerts biological effects through binding to specific 5-HT receptors on the cell membrane. Previous studies have shown reduced intestinal 5-HT level in loperamide hydrochloride-induced constipation models (32, 42). In contrast, increased *TPH1* expression and elevated 5-HT level have been observed in rectal biopsy samples from patients with constipation (43). Moreover, in patient populations with constipation-predominant irritable bowel syndrome (IBS-C) and functional constipation (FC), a negative correlation between defecation frequency and 5-HT concentration has been identified (44). The discrepancies among these results may be attributed to the complex effects of 5-HT on gastrointestinal motor activity. In the present study, a constipation mouse model was established by transplanting fecal bacterial suspension from patients with STC. The results showed a significant increase in colonic 5-HT levels in constipated mice, consistent with previous findings (24). In addition, the results indicated that compared with the control group, the relative expression of *TPH1* and *Piezo1* in the colon of constipated mice was significantly elevated. However, *L. acidophilus* treatment effectively regulated and normalized the expression of *TPH1* and *Piezo1*, and these findings were corroborated by *in vitro* cell experiments. These findings could be attributed to both the activation and desensitization of 5-HT receptors and the characteristics of the experimental STC model employed.

## Conclusion

STC is an intractable constipation with unknown etiology and uncertain pathogenesis, significantly impacting patients’ physical and mental well-being as well as their overall quality of life. In this study, a humanized constipation mouse model was established via FMT, and the therapeutic effect of *L. acidophilus* on constipation was demonstrated by analyzing the defecation parameters. Through integrated *in vivo* and *in vitro* analyses, we elucidated the mechanisms by which *L. acidophilus* alleviates constipation. Our findings demonstrate that it not only repairs the intestinal barrier, but also modulates the 5-HT pathway by regulating the expression of *TPH1* and *Piezo1*. Additionally, metagenomic sequencing revealed that *L. acidophilus* remodels the gut microbiota structure and interaction network. In summary, *L. acidophilus* exerts its therapeutic effect through a multi-faceted action on the gut barrier, serotonin signaling, and microbial ecology.

## Data Availability

The datasets presented in this study can be found in online repositories. The names of the repository/repositories and accession number(s) can be found at: https://www.ebi.ac.uk/arrayexpress/, E-MTAB-16505.

## Footnotes

1
https://genome.ucsc.edu
